# Supplementary material for: Obesity and mortality after the first ischemic stroke: Is obesity paradox real?
Source: PLoS One. 2021 Feb 10;16(2):e0246877. doi: 10.1371/journal.pone.0246877 (PMC7875337; doi:10.1371/journal.pone.0246877)
Supplement: S1 Text — (DOCX) [file pone.0246877.s001.docx]

**Obesity and Mortality after the First Ischemic Stroke: Is Obesity Paradox Real?**

SUPPLEMENTAL MATERIALS

1. [Table A. ICD-9-CM/ICD-10-CM Codes used to ischemic stroke and risk factors/comorbidities](#_Table_S1._ICD-9-CM/ICD-10-CM)
2. [Table B. CPT codes used for MRI Brain](#_Table_S2._CPT)
3. [Table C. Data elements from GNSIS database included in the study](#_Table_S3._Data)
4. [Table D. Comparison of Age, Sex and NIHSS between excluded patients with missing BMI and patients included in the analysis](#_Table_S4._Comparison)
5. [Table E. p-values for pairwise Log-rank test for survival at one year for four BMI categories](#_Table_S5._P-values)
6. [Table F. p-values for pairwise log-rank test for survival at one year for three BMI categories](#_Table_S6._P-values)
7. [Table G. Multivariate stratified Cox proportional hazards model on one-year mortality for patients with NIHSS data (N=1782)](#_Table_S7._Multivariate)
8. [Table H. Assessment of Schoenfeld residuals for the stratified Cox model for all patients in the study](#_Table_S9._Assessment)
9. [Table I. Assessment of Schoenfeld residuals for stratified Cox model patients with NIHSS data](#_Table_S10._Assessment)
10. [Table J. Multivariate logistic regression on one-year mortality for patients with at least one-year follow-up data](#_Table_J._Multivariate)
11. [Table K. Summary of different studies regarding Obesity and Stroke Mortality](#_Table_S11._Summary)
12. [Table L. New diagnoses of comorbidities after index stroke event for the overall study population](#_Table_S12._New)
13. [Table M. New diagnoses of comorbidities after index stroke event for the three BMI groups](#_Table_S13._New)
14. [Figure A. Flow-chart of inclusion-exclusion of subjects in the study](#_Figure_S1._Flow-chart)
15. [Figure B. Margin plots of missing BMI with respect to Age, Sex and NIHSS](#_Figure_S2._Margin)

# Table A. ICD-9-CM/ICD-10-CM Codes used to ischemic stroke and risk factors/comorbidities

| Condition | ICD-9 | ICD-10 |
| --- | --- | --- |
| Ischemic Stroke | 433.01, 433.11, 433.21, 433.31, 433.81, 433.91, 434.01, 434.11, 434.91 | I63, I63.0, I63.00, I63.01, I63.011, I63.012, I63.013, I63.019, I63.02, I63.03, I63.031, I63.032, I63.033, I63.039, I63.09, I63.1, I63.10, I63.11, I63.111, I63.112, I63.113, I63.119, I63.12, I63.13, I63.131, I63.132, I63.133, I63.139, I63.19, I63.2, I63.20, I63.21, I63.211, I63.212, I63.213, I63.219, I63.22, I63.23, I63.231, I63.232, I63.233, I63.239, I63.29, I63.3, I63.30, I63.31, I63.311, I63.312, I63.313, I63.319, I63.32, I63.321, I63.322, I63.323, I63.329, I63.33, I63.331, I63.332, I63.333, I63.339, I63.34, I63.341, I63.342, I63.343, I63.349, I63.39, I63.4, I63.40, I63.41, I63.411, I63.412, I63.413, I63.419, I63.42, I63.421, I63.422, I63.423, I63.429, I63.43, I63.431, I63.432, I63.433, I63.439, I63.44, I63.441, I63.442, I63.443, I63.449, I63.49, I63.5, I63.50, I63.51, I63.511, I63.512, I63.513, I63.519, I63.52, I63.521, I63.522, I63.523, I63.529, I63.53, I63.531, I63.532, I63.533, I63.539, I63.54, I63.541, I63.542, I63.543, I63.549, I63.59, I63.6, I63.8, I63.9 |
| Hemorrhagic Stroke | 431, 432.9, 430 | I61, I61.0, I61.1, I61.2, I61.3, I61.4, I61.5, I61.6, I61.8, I61.9, I62.9, I60, I60.0, I60.00, I60.01, I60.02, I60.1, I60.10, I60.11, I60.12, I60.2, I60.3, I60.30, I60.31, I60.32, I60.4, I60.5, I60.50, I60.51, I60.52, I60.6, I60.7, I60.8, I60.9 |
| Atrial Fibrillation | 427.3, 427.31 | I48, I48.0, I48.1, I48.11, I48.19, I48.2, I48.20, I48.21, I48.9, I48.91 |
| Atrial Flutter | 427.32 | I48.3, I48.4, I48.92 |
| Hypertension | 401, 401.0, 401.1, 401.9, 402, 402.0, 402.00, 402.01, 402.1, 402.10, 402.11, 402.9, 402.90, 402.91, 403, 403.0, 403.00, 403.01, 403.1, 403.10, 403.11, 403.9, 403.90, 403.91, 404, 404.0, 404.00, 404.01, 404.02, 404.03, 404.1, 404.10, 404.11, 404.12, 404.13, 404.9, 404.90, 404.91, 404.92, 404.93, 405, 405.0, 405.01, 405.09, 405.1, 405.11, 405.19, 405.9, 405.91, 405.99 | I10, I11, I11.0, I11.9, I12, I12.0, I12.9, I13, I13.0, I13.1, I13.10, I13.11, I13.2, I15, I15.0, I15.1, I15.2, I15.8, I15.9, I16, I16.0, I16.1, I16.9 |
| Myocardial Infarction | 410, 410.0, 410.00, 410.01, 410.02, 410.1, 410.10, 410.11, 410.12, 410.2, 410.20, 410.21, 410.22, 410.3, 410.30, 410.31, 410.32, 410.4, 410.40, 410.41, 410.42, 410.5, 410.50, 410.51, 410.52, 410.6, 410.60, 410.61, 410.62, 410.7, 410.70, 410.71, 410.72, 410.8, 410.80, 410.81, 410.82, 410.9, 410.90, 410.91, 410.92, 412, 429.79 | I21, I21.0, I21.01, I21.02, I21.09, I21.1, I21.11, I21.19, I21.2, I21.21, I21.29, I21.3, I21.4, I21.9, I21.A, I21.A1, I21.A9, I22, I22.0, I22.1, I22.2, I22.8, I22.9, I23, I23.0, I23.1, I23.2, I23.3, I23.4, I23.5, I23.6, I23.7, I23.8, I25.2 |
| Diabetes Mellitus | 249, 249.0, 249.00, 249.01, 249.1, 249.10, 249.11, 249.2, 249.20, 249.21, 249.3, 249.30, 249.31, 249.4, 249.40, 249.41, 249.5, 249.50, 249.51, 249.6, 249.60, 249.61, 249.7, 249.70, 249.71, 249.8, 249.80, 249.81, 249.9, 249.90, 249.91, 250, 250.0, 250.00, 250.01, 250.02, 250.03, 250.1, 250.10, 250.11, 250.12, 250.13, 250.2, 250.20, 250.21, 250.22, 250.23, 250.3, 250.30, 250.31, 250.32, 250.33, 250.4, 250.40, 250.41, 250.42, 250.43, 250.5, 250.50, 250.51, 250.52, 250.53, 250.6, 250.60, 250.61, 250.62, 250.63, 250.7, 250.70, 250.71, 250.72, 250.73, 250.8, 250.80, 250.81, 250.82, 250.83, 250.9, 250.90, 250.91, 250.92, 250.93, 357.2, 362.01, 362.02, 362.03, 362.04, 362.05, 362.06, 362.07, 366.41 | E10 , E10.1 , E10.10 , E10.11 , E10.2 , E10.21 , E10.22 , E10.29 , E10.3 , E10.31 , E10.311 , E10.319 , E10.32 , E10.321 , E10.3211 , E10.3212 , E10.3213 , E10.3219 , E10.329 , E10.3291 , E10.3292 , E10.3293 , E10.3299 , E10.33 , E10.331 , E10.3311 , E10.3312 , E10.3313 , E10.3319 , E10.339 , E10.3391 , E10.3392 , E10.3393 , E10.3399 , E10.34 , E10.341 , E10.3411 , E10.3412 , E10.3413 , E10.3419 , E10.349 , E10.3491 , E10.3492 , E10.3493 , E10.3499 , E10.35 , E10.351 , E10.3511 , E10.3512 , E10.3513 , E10.3519 , E10.352 , E10.3521 , E10.3522 , E10.3523 , E10.3529 , E10.353 , E10.3531 , E10.3532 , E10.3533 , E10.3539 , E10.354 , E10.3541 , E10.3542 , E10.3543 , E10.3549 , E10.355 , E10.3551 , E10.3552 , E10.3553 , E10.3559 , E10.359 , E10.3591 , E10.3592 , E10.3593 , E10.3599 , E10.36 , E10.37 , E10.37X1 , E10.37X2 , E10.37X3 , E10.37X9 , E10.39 , E10.4 , E10.40 , E10.41 , E10.42 , E10.43 , E10.44 , E10.49 , E10.5 , E10.51 , E10.52 , E10.59 , E10.6 , E10.61 , E10.610 , E10.618 , E10.62 , E10.620 , E10.621 , E10.622 , E10.628 , E10.63 , E10.630 , E10.638 , E10.64 , E10.641 , E10.649 , E10.65 , E10.69 , E10.8 , E10.9 , E11 , E11.0 , E11.00 , E11.01 , E11.1 , E11.10 , E11.11 , E11.2 , E11.21 , E11.22 , E11.29 , E11.3 , E11.31 , E11.311 , E11.319 , E11.32 , E11.321 , E11.3211 , E11.3212 , E11.3213 , E11.3219 , E11.329 , E11.3291 , E11.3292 , E11.3293 , E11.3299 , E11.33 , E11.331 , E11.3311 , E11.3312 , E11.3313 , E11.3319 , E11.339 , E11.3391 , E11.3392 , E11.3393 , E11.3399 , E11.34 , E11.341 , E11.3411 , E11.3412 , E11.3413 , E11.3419 , E11.349 , E11.3491 , E11.3492 , E11.3493 , E11.3499 , E11.35 , E11.351 , E11.3511 , E11.3512 , E11.3513 , E11.3519 , E11.352 , E11.3521 , E11.3522 , E11.3523 , E11.3529 , E11.353 , E11.3531 , E11.3532 , E11.3533 , E11.3539 , E11.354 , E11.3541 , E11.3542 , E11.3543 , E11.3549 , E11.355 , E11.3551 , E11.3552 , E11.3553 , E11.3559 , E11.359 , E11.3591 , E11.3592 , E11.3593 , E11.3599 , E11.36 , E11.37 , E11.37X1 , E11.37X2 , E11.37X3 , E11.37X9 , E11.39 , E11.4 , E11.40 , E11.41 , E11.42 , E11.43 , E11.44 , E11.49 , E11.5 , E11.51 , E11.52 , E11.59 , E11.6 , E11.61 , E11.610 , E11.618 , E11.62 , E11.620 , E11.621 , E11.622 , E11.628 , E11.63 , E11.630 , E11.638 , E11.64 , E11.641 , E11.649 , E11.65 , E11.69 , E11.8 , E11.9 , E08 , E08.0 , E08.00 , E08.01 , E08.1 , E08.10 , E08.11 , E08.2 , E08.21 , E08.22 , E08.29 , E08.3 , E08.31 , E08.311 , E08.319 , E08.32 , E08.321 , E08.3211 , E08.3212 , E08.3213 , E08.3219 , E08.329 , E08.3291 , E08.3292 , E08.3293 , E08.3299 , E08.33 , E08.331 , E08.3311 , E08.3312 , E08.3313 , E08.3319 , E08.339 , E08.3391 , E08.3392 , E08.3393 , E08.3399 , E08.34 , E08.341 , E08.3411 , E08.3412 , E08.3413 , E08.3419 , E08.349 , E08.3491 , E08.3492 , E08.3493 , E08.3499 , E08.35 , E08.351 , E08.3511 , E08.3512 , E08.3513 , E08.3519 , E08.352 , E08.3521 , E08.3522 , E08.3523 , E08.3529 , E08.353 , E08.3531 , E08.3532 , E08.3533 , E08.3539 , E08.354 , E08.3541 , E08.3542 , E08.3543 , E08.3549 , E08.355 , E08.3551 , E08.3552 , E08.3553 , E08.3559 , E08.359 , E08.3591 , E08.3592 , E08.3593 , E08.3599 , E08.36 , E08.37 , E08.37X1 , E08.37X2 , E08.37X3 , E08.37X9 , E08.39 , E08.4 , E08.40 , E08.41 , E08.42 , E08.43 , E08.44 , E08.49 , E08.5 , E08.51 , E08.52 , E08.59 , E08.6 , E08.61 , E08.610 , E08.618 , E08.62 , E08.620 , E08.621 , E08.622 , E08.628 , E08.63 , E08.630 , E08.638 , E08.64 , E08.641 , E08.649 , E08.65 , E08.69 , E08.8 , E08.9 , E09 , E09.0 , E09.00 , E09.01 , E09.1 , E09.10 , E09.11 , E09.2 , E09.21 , E09.22 , E09.29 , E09.3 , E09.31 , E09.311 , E09.319 , E09.32 , E09.321 , E09.3211 , E09.3212 , E09.3213 , E09.3219 , E09.329 , E09.3291 , E09.3292 , E09.3293 , E09.3299 , E09.33 , E09.331 , E09.3311 , E09.3312 , E09.3313 , E09.3319 , E09.339 , E09.3391 , E09.3392 , E09.3393 , E09.3399 , E09.34 , E09.341 , E09.3411 , E09.3412 , E09.3413 , E09.3419 , E09.349 , E09.3491 , E09.3492 , E09.3493 , E09.3499 , E09.35 , E09.351 , E09.3511 , E09.3512 , E09.3513 , E09.3519 , E09.352 , E09.3521 , E09.3522 , E09.3523 , E09.3529 , E09.353 , E09.3531 , E09.3532 , E09.3533 , E09.3539 , E09.354 , E09.3541 , E09.3542 , E09.3543 , E09.3549 , E09.355 , E09.3551 , E09.3552 , E09.3553 , E09.3559 , E09.359 , E09.3591 , E09.3592 , E09.3593 , E09.3599 , E09.36 , E09.37 , E09.37X1 , E09.37X2 , E09.37X3 , E09.37X9 , E09.39 , E09.4 , E09.40 , E09.41 , E09.42 , E09.43 , E09.44 , E09.49 , E09.5 , E09.51 , E09.52 , E09.59 , E09.6 , E09.61 , E09.610 , E09.618 , E09.62 , E09.620 , E09.621 , E09.622 , E09.628 , E09.63 , E09.630 , E09.638 , E09.64 , E09.641 , E09.649 , E09.65 , E09.69 , E09.8 , E09.9 , E13 , E13.0 , E13.00 , E13.01 , E13.1 , E13.10 , E13.11 , E13.2 , E13.21 , E13.22 , E13.29 , E13.3 , E13.31 , E13.311 , E13.319 , E13.32 , E13.321 , E13.3211 , E13.3212 , E13.3213 , E13.3219 , E13.329 , E13.3291 , E13.3292 , E13.3293 , E13.3299 , E13.33 , E13.331 , E13.3311 , E13.3312 , E13.3313 , E13.3319 , E13.339 , E13.3391 , E13.3392 , E13.3393 , E13.3399 , E13.34 , E13.341 , E13.3411 , E13.3412 , E13.3413 , E13.3419 , E13.349 , E13.3491 , E13.3492 , E13.3493 , E13.3499 , E13.35 , E13.351 , E13.3511 , E13.3512 , E13.3513 , E13.3519 , E13.352 , E13.3521 , E13.3522 , E13.3523 , E13.3529 , E13.353 , E13.3531 , E13.3532 , E13.3533 , E13.3539 , E13.354 , E13.3541 , E13.3542 , E13.3543 , E13.3549 , E13.355 , E13.3551 , E13.3552 , E13.3553 , E13.3559 , E13.359 , E13.3591 , E13.3592 , E13.3593 , E13.3599 , E13.36 , E13.37 , E13.37X1 , E13.37X2 , E13.37X3 , E13.37X9 , E13.39 , E13.4 , E13.40 , E13.41 , E13.42 , E13.43 , E13.44 , E13.49 , E13.5 , E13.51 , E13.52 , E13.59 , E13.6 , E13.61 , E13.610 , E13.618 , E13.62 , E13.620 , E13.621 , E13.622 , E13.628 , E13.63 , E13.630 , E13.638 , E13.64 , E13.641 , E13.649 , E13.65 , E13.69 , E13.8 , E13.9 |
| Dyslipidemia | 272.0, 272.1, 272.2, 272.3, 272.4, 272.5 | E78.0, E78.00, E78.01, E78.1, E78.2, E78.3, E78.4, E78.41, E78.49, E78.5, E78.6 |
| Heart failure | 402.01, 402.11, 402.91, 404.01, 404.03, 404.11, 404.13, 404.91, 404.93, 428, 428.0, 428.1, 428.2, 428.20, 428.21, 428.22, 428.23, 428.3, 428.30, 428.31, 428.32, 428.33, 428.4, 428.40, 428.41, 428.42, 428.43, 428.9 | I11.0, I13.0, I13.2, I50, I50.1, I50.2, I50.20, I50.21, I50.22, I50.23, I50.3, I50.30, I50.31, I50.32, I50.33, I50.4, I50.40, I50.41, I50.42, I50.43, I50.8, I50.81, I50.810, I50.811, I50.812, I50.813, I50.814, I50.82, I50.83, I50.84, I50.89, I50.9 |
| Hypercoagulable states | 289.81, 289.82 | D68.5, D68.51, D68.52, D68.59, D68.6, D68.61, D68.62, D68.69 |
| Chronic liver disease | 070.32 , 070.33, 070.54, 070.9 , 570, 571.0, 571.2, 571.3, 571.4, 571.40, 571.41, 571.42, 571.49, 571.5, 571.6, 571.8, 571.9, 573.3 , 573.4,  070.22, 070.23, 070.44 , 070.6, 456.0, 456.1, 456.2, 456.20, 456.21, 567.23, 572.1, 572.2, 572.3, 572.4, 572.8 | B18, B18.0, B18.1, B18.2, B18.8, B18.9, K70.0, K70.1, K70.10, K70.2, K70.3, K70.30, K70.9, K71.3, K71.4, K71.5, K71.7, K73, K73.0, K73.1, K73.2, K73.8, K73.9, K74, K74.0, K74.1, K74.2, K74.3, K74.4, K74.5, K74.6, K74.60, K74.69, K76.0, K76.2, K76.3, K76.4, K76.8, K76.9, Z94.4  I85.0, I86.4, K70.11, K70.31, K70.4, K70.40, K70.41, K71.1, K72.1, K72.9, K76.5, K76.6, K76.7 |
| Chronic lung disease  (COPD, Asthma, Occupational lung diseases) | 490, 491, 491.0, 491.1, 491.2, 491.20, 491.21, 491.22, 491.8, 491.9, 492, 492.0, 492.8, 493, 493.0, 493.00, 493.01, 493.02, 493.1, 493.10, 493.11, 493.12, 493.2, 493.20, 493.21, 493.22, 493.8, 493.81, 493.82, 493.9, 493.90, 493.91, 493.92, 494, 494.0, 494.1, 495, 495.0, 495.1, 495.2, 495.3, 495.4, 495.5, 495.6, 495.7, 495.8, 495.9, 496, 500, 501, 502, 503, 504, 505, 506.4, 508.1 | J40, J41, J41.0, J41.1, J41.8, J42, J43, J43.0, J43.1, J43.2, J43.8, J43.9, J44, J44.0, J44.1, J44.9, J45, J45.2, J45.20, J45.21, J45.22, J45.3, J45.30, J45.31, J45.32, J45.4, J45.40, J45.41, J45.42, J45.5, J45.50, J45.51, J45.52, J45.9, J45.90, J45.901, J45.902, J45.909, J45.99, J45.990, J45.991, J45.998, J47, J47.0, J47.1, J47.9, J60, J61, J62, J62.0, J62.8, J63, J63.0, J63.1, J63.2, J63.3, J63.4, J63.5, J63.6, J64, J65, J66, J66.0, J66.1, J66.2, J66.8, J67, J67.0, J67.1, J67.2, J67.3, J67.4, J67.5, J67.6, J67.7, J67.8, J67.9, J68.4, J70.1, J70.3 |
| Rheumatic diseases | 710, 710.0, 710.1, 710.2, 710.3, 710.4, 710.5, 710.8, 710.9, 714, 714.0, 714.1, 714.2, 714.4, 714.8, 714.81, 714.89, 714.9, 725 | M05, M05.0, M05.00, M05.01, M05.011, M05.012, M05.019, M05.02, M05.021, M05.022, M05.029, M05.03, M05.031, M05.032, M05.039, M05.04, M05.041, M05.042, M05.049, M05.05, M05.051, M05.052, M05.059, M05.06, M05.061, M05.062, M05.069, M05.07, M05.071, M05.072, M05.079, M05.09, M05.1, M05.10, M05.11, M05.111, M05.112, M05.119, M05.12, M05.121, M05.122, M05.129, M05.13, M05.131, M05.132, M05.139, M05.14, M05.141, M05.142, M05.149, M05.15, M05.151, M05.152, M05.159, M05.16, M05.161, M05.162, M05.169, M05.17, M05.171, M05.172, M05.179, M05.19, M05.2, M05.20, M05.21, M05.211, M05.212, M05.219, M05.22, M05.221, M05.222, M05.229, M05.23, M05.231, M05.232, M05.239, M05.24, M05.241, M05.242, M05.249, M05.25, M05.251, M05.252, M05.259, M05.26, M05.261, M05.262, M05.269, M05.27, M05.271, M05.272, M05.279, M05.29, M05.3, M05.30, M05.31, M05.311, M05.312, M05.319, M05.32, M05.321, M05.322, M05.329, M05.33, M05.331, M05.332, M05.339, M05.34, M05.341, M05.342, M05.349, M05.35, M05.351, M05.352, M05.359, M05.36, M05.361, M05.362, M05.369, M05.37, M05.371, M05.372, M05.379, M05.39, M05.4, M05.40, M05.41, M05.411, M05.412, M05.419, M05.42, M05.421, M05.422, M05.429, M05.43, M05.431, M05.432, M05.439, M05.44, M05.441, M05.442, M05.449, M05.45, M05.451, M05.452, M05.459, M05.46, M05.461, M05.462, M05.469, M05.47, M05.471, M05.472, M05.479, M05.49, M05.5, M05.50, M05.51, M05.511, M05.512, M05.519, M05.52, M05.521, M05.522, M05.529, M05.53, M05.531, M05.532, M05.539, M05.54, M05.541, M05.542, M05.549, M05.55, M05.551, M05.552, M05.559, M05.56, M05.561, M05.562, M05.569, M05.57, M05.571, M05.572, M05.579, M05.59, M05.6, M05.60, M05.61, M05.611, M05.612, M05.619, M05.62, M05.621, M05.622, M05.629, M05.63, M05.631, M05.632, M05.639, M05.64, M05.641, M05.642, M05.649, M05.65, M05.651, M05.652, M05.659, M05.66, M05.661, M05.662, M05.669, M05.67, M05.671, M05.672, M05.679, M05.69, M05.7, M05.70, M05.71, M05.711, M05.712, M05.719, M05.72, M05.721, M05.722, M05.729, M05.73, M05.731, M05.732, M05.739, M05.74, M05.741, M05.742, M05.749, M05.75, M05.751, M05.752, M05.759, M05.76, M05.761, M05.762, M05.769, M05.77, M05.771, M05.772, M05.779, M05.79, M05.8, M05.80, M05.81, M05.811, M05.812, M05.819, M05.82, M05.821, M05.822, M05.829, M05.83, M05.831, M05.832, M05.839, M05.84, M05.841, M05.842, M05.849, M05.85, M05.851, M05.852, M05.859, M05.86, M05.861, M05.862, M05.869, M05.87, M05.871, M05.872, M05.879, M05.89, M05.9, M06, M06.0, M06.00, M06.01, M06.011, M06.012, M06.019, M06.02, M06.021, M06.022, M06.029, M06.03, M06.031, M06.032, M06.039, M06.04, M06.041, M06.042, M06.049, M06.05, M06.051, M06.052, M06.059, M06.06, M06.061, M06.062, M06.069, M06.07, M06.071, M06.072, M06.079, M06.08, M06.09, M06.1, M06.2, M06.20, M06.21, M06.211, M06.212, M06.219, M06.22, M06.221, M06.222, M06.229, M06.23, M06.231, M06.232, M06.239, M06.24, M06.241, M06.242, M06.249, M06.25, M06.251, M06.252, M06.259, M06.26, M06.261, M06.262, M06.269, M06.27, M06.271, M06.272, M06.279, M06.28, M06.29, M06.3, M06.30, M06.31, M06.311, M06.312, M06.319, M06.32, M06.321, M06.322, M06.329, M06.33, M06.331, M06.332, M06.339, M06.34, M06.341, M06.342, M06.349, M06.35, M06.351, M06.352, M06.359, M06.36, M06.361, M06.362, M06.369, M06.37, M06.371, M06.372, M06.379, M06.38, M06.39, M06.4, M06.8, M06.80, M06.81, M06.811, M06.812, M06.819, M06.82, M06.821, M06.822, M06.829, M06.83, M06.831, M06.832, M06.839, M06.84, M06.841, M06.842, M06.849, M06.85, M06.851, M06.852, M06.859, M06.86, M06.861, M06.862, M06.869, M06.87, M06.871, M06.872, M06.879, M06.88, M06.89, M06.9, M31.5, M32, M32.0, M32.1, M32.10, M32.11, M32.12, M32.13, M32.14, M32.15, M32.19, M32.8, M32.9, M33, M33.0, M33.00, M33.01, M33.02, M33.03, M33.09, M33.1, M33.10, M33.11, M33.12, M33.13, M33.19, M33.2, M33.20, M33.21, M33.22, M33.29, M33.9, M33.90, M33.91, M33.92, M33.93, M33.99, M34, M34.0, M34.1, M34.2, M34.8, M34.81, M34.82, M34.83, M34.89, M34.9, M35.1, M35.3, M36.0 |
| Chronic kidney disease | 403.00, 403.01, 403.10, 403.11, 403.90, 403.91, 404.00, 404.01, 404.02, 404.03, 404.10, 404.11, 404.12, 404.13, 404.90, 404.91, 404.92, 404.93, 585, 585.1, 585.2, 585.3, 585.4, 585.5, 585.6, 585.9, 586 | I12, I12.0, I12.9, I13, I13.0, I13.1, I13.10, I13.11, I13.2, N18, N18.1, N18.2, N18.3, N18.4, N18.5, N18.6, N18.9, N19, Z49.0, Z94.0, Z99.2 |
| Peripheral vascular disease | 440, 440.0, 440.1, 440.2, 440.20, 440.21, 440.22, 440.23, 440.24, 440.29, 440.3, 440.30, 440.31, 440.32, 440.4, 440.8, 440.9, 441, 441.0, 441.00, 441.01, 441.02, 441.03, 441.1, 441.2, 441.3, 441.4, 441.5, 441.6, 441.7, 441.9, 443.0, 443.1, 443.8, 443.81, 443.82, 443.89, 443.9, 447.1, 557, 557.0, 557.1, 557.9 | I70, I70.0, I70.1, I70.2, I70.20, I70.201, I70.202, I70.203, I70.208, I70.209, I70.21, I70.211, I70.212, I70.213, I70.218, I70.219, I70.22, I70.221, I70.222, I70.223, I70.228, I70.229, I70.23, I70.231, I70.232, I70.233, I70.234, I70.235, I70.238, I70.239, I70.24, I70.241, I70.242, I70.243, I70.244, I70.245, I70.248, I70.249, I70.25, I70.26, I70.261, I70.262, I70.263, I70.268, I70.269, I70.29, I70.291, I70.292, I70.293, I70.298, I70.299, I70.3, I70.30, I70.301, I70.302, I70.303, I70.308, I70.309, I70.31, I70.311, I70.312, I70.313, I70.318, I70.319, I70.32, I70.321, I70.322, I70.323, I70.328, I70.329, I70.33, I70.331, I70.332, I70.333, I70.334, I70.335, I70.338, I70.339, I70.34, I70.341, I70.342, I70.343, I70.344, I70.345, I70.348, I70.349, I70.35, I70.36, I70.361, I70.362, I70.363, I70.368, I70.369, I70.39, I70.391, I70.392, I70.393, I70.398, I70.399, I70.4, I70.40, I70.401, I70.402, I70.403, I70.408, I70.409, I70.41, I70.411, I70.412, I70.413, I70.418, I70.419, I70.42, I70.421, I70.422, I70.423, I70.428, I70.429, I70.43, I70.431, I70.432, I70.433, I70.434, I70.435, I70.438, I70.439, I70.44, I70.441, I70.442, I70.443, I70.444, I70.445, I70.448, I70.449, I70.45, I70.46, I70.461, I70.462, I70.463, I70.468, I70.469, I70.49, I70.491, I70.492, I70.493, I70.498, I70.499, I70.5, I70.50, I70.501, I70.502, I70.503, I70.508, I70.509, I70.51, I70.511, I70.512, I70.513, I70.518, I70.519, I70.52, I70.521, I70.522, I70.523, I70.528, I70.529, I70.53, I70.531, I70.532, I70.533, I70.534, I70.535, I70.538, I70.539, I70.54, I70.541, I70.542, I70.543, I70.544, I70.545, I70.548, I70.549, I70.55, I70.56, I70.561, I70.562, I70.563, I70.568, I70.569, I70.59, I70.591, I70.592, I70.593, I70.598, I70.599, I70.6, I70.60, I70.601, I70.602, I70.603, I70.608, I70.609, I70.61, I70.611, I70.612, I70.613, I70.618, I70.619, I70.62, I70.621, I70.622, I70.623, I70.628, I70.629, I70.63, I70.631, I70.632, I70.633, I70.634, I70.635, I70.638, I70.639, I70.64, I70.641, I70.642, I70.643, I70.644, I70.645, I70.648, I70.649, I70.65, I70.66, I70.661, I70.662, I70.663, I70.668, I70.669, I70.69, I70.691, I70.692, I70.693, I70.698, I70.699, I70.7, I70.70, I70.701, I70.702, I70.703, I70.708, I70.709, I70.71, I70.711, I70.712, I70.713, I70.718, I70.719, I70.72, I70.721, I70.722, I70.723, I70.728, I70.729, I70.73, I70.731, I70.732, I70.733, I70.734, I70.735, I70.738, I70.739, I70.74, I70.741, I70.742, I70.743, I70.744, I70.745, I70.748, I70.749, I70.75, I70.76, I70.761, I70.762, I70.763, I70.768, I70.769, I70.79, I70.791, I70.792, I70.793, I70.798, I70.799, I70.8, I70.9, I70.90, I70.91, I70.92, I73, I73.0, I73.00, I73.01, I73.1, I73.8, I73.81, I73.89, I73.9, I77.1, I79.0 |
| Neoplasm | 140.0, 140.1, 140.4, 140.5, 140.6, 140.8, 140.9, 141.0, 141.1, 141.2, 141.3, 141.4, 141.5, 141.6, 141.8, 141.9, 142.0, 142.1, 142.2, 142.8, 142.9, 143.0, 143.1, 143.8, 143.9, 144.0, 144.1, 144.8, 144.9, 145.0, 145.1, 145.2, 145.3, 145.4, 145.5, 145.6, 145.8, 145.9, 146.0, 146.1, 146.2, 146.3, 146.4, 146.5, 146.6, 146.7, 146.8, 146.9, 147.0, 147.1, 147.2, 147.3, 147.8, 147.9, 148.0, 148.1, 148.2, 148.3, 148.8, 148.9, 149.0, 149.8, 149.9, 150.0, 150.1, 150.2, 150.3, 150.4, 150.5, 150.8, 150.9, 151.0, 151.1, 151.2, 151.3, 151.4, 151.5, 151.6, 151.8, 151.9, 152.0, 152.1, 152.2, 152.3, 152.8, 152.9, 153, 153.0, 153.1, 153.2, 153.3, 153.4, 153.5, 153.6, 153.7, 153.8, 153.9, 154, 154.0, 154.1, 154.2, 154.3, 154.8, 155.0, 155.1, 155.2, 156.0, 156.1, 156.2, 156.8, 156.9, 157, 157.0, 157.1, 157.2, 157.3, 157.4, 157.8, 157.9, 158, 158.0, 158.8, 158.9, 159, 159.0, 159.1, 159.8, 159.9, 160.0, 160.1, 160.2, 160.3, 160.4, 160.5, 160.9, 161.0, 161.1, 161.2, 161.3, 161.8, 161.9, 162, 162.0, 162.2, 162.3, 162.4, 162.5, 162.8, 162.9, 163.0, 163.1, 163.8, 163.9, 164.0, 164.1, 164.2, 164.3, 164.8, 164.9, 165.8, 165.9, 170.0, 170.1, 170.2, 170.3, 170.4, 170.5, 170.6, 170.7, 170.8, 170.9, 171.0, 171.2, 171.3, 171.4, 171.5, 171.6, 171.7, 171.8, 171.9, 172.0, 172.1, 172.2, 172.3, 172.4, 172.5, 172.6, 172.7, 172.8, 172.9, 173.0, 173.00, 173.01, 173.02, 173.09, 173.1, 173.10, 173.11, 173.12, 173.19, 173.2, 173.20, 173.21, 173.22, 173.29, 173.3, 173.30, 173.31, 173.32, 173.39, 173.4, 173.40, 173.41, 173.42, 173.49, 173.5, 173.50, 173.51, 173.52, 173.59, 173.6, 173.60, 173.61, 173.62, 173.69, 173.7, 173.70, 173.71, 173.72, 173.79, 173.8, 173.80, 173.81, 173.82, 173.89, 173.9, 173.90, 173.91, 173.92, 173.99, 174, 174.0, 174.1, 174.2, 174.3, 174.4, 174.5, 174.6, 174.8, 174.9, 175.0, 175.9, 176.0, 176.1, 176.2, 176.3, 176.4, 176.8, 176.9, 179, 180.0, 180.1, 180.8, 180.9, 181, 182.0, 182.1, 182.8, 183, 183.0, 183.2, 183.4, 183.5, 183.8, 183.9, 184.0, 184.1, 184.2, 184.3, 184.4, 184.8, 184.9, 185, 186.0, 186.9, 187.1, 187.2, 187.3, 187.4, 187.6, 187.7, 187.8, 187.9, 188.0, 188.1, 188.2, 188.3, 188.4, 188.5, 188.6, 188.7, 188.8, 188.9, 189.0, 189.1, 189.2, 189.3, 189.4, 189.8, 189.9, 190.0, 190.1, 190.2, 190.3, 190.4, 190.5, 190.6, 190.7, 190.8, 190.9, 191.0, 191.1, 191.2, 191.3, 191.4, 191.5, 191.6, 191.7, 191.8, 191.9, 192.0, 192.1, 192.2, 192.3, 192.8, 192.9, 193, 194.0, 194.1, 194.3, 194.4, 194.5, 194.6, 194.8, 194.9, 195.0, 195.1, 195.2, 195.3, 195.4, 195.5, 195.8, 196.0, 196.1, 196.2, 196.3, 196.5, 196.6, 196.8, 196.9, 197.0, 197.1, 197.2, 197.3, 197.4, 197.5, 197.6, 197.7, 197.8, 198.0, 198.1, 198.2, 198.3, 198.4, 198.5, 198.6, 198.7, 198.81, 198.82, 198.89, 199, 199.0, 199.1, 199.2, 200, 200.00, 200.01, 200.02, 200.03, 200.04, 200.05, 200.06, 200.08, 200.10, 200.11, 200.12, 200.13, 200.14, 200.15, 200.16, 200.18, 200.20, 200.21, 200.22, 200.23, 200.24, 200.27, 200.28, 200.30, 200.31, 200.32, 200.33, 200.34, 200.35, 200.37, 200.38, 200.40, 200.41, 200.42, 200.43, 200.44, 200.45, 200.47, 200.48, 200.50, 200.51, 200.53, 200.57, 200.58, 200.60, 200.61, 200.62, 200.63, 200.64, 200.65, 200.68, 200.70, 200.71, 200.72, 200.73, 200.74, 200.75, 200.77, 200.78, 200.8, 200.80, 200.81, 200.82, 200.83, 200.88, 201.00, 201.01, 201.10, 201.12, 201.20, 201.21, 201.25, 201.26, 201.40, 201.42, 201.43, 201.44, 201.45, 201.47, 201.48, 201.50, 201.51, 201.52, 201.53, 201.54, 201.58, 201.60, 201.61, 201.63, 201.64, 201.68, 201.70, 201.71, 201.72, 201.73, 201.74, 201.75, 201.78, 201.9, 201.90, 201.91, 201.92, 201.93, 201.94, 201.95, 201.96, 201.97, 201.98, 202.00, 202.01, 202.02, 202.03, 202.04, 202.05, 202.06, 202.07, 202.08, 202.10, 202.11, 202.12, 202.13, 202.14, 202.15, 202.16, 202.17, 202.18, 202.20, 202.21, 202.28, 202.30, 202.31, 202.32, 202.33, 202.34, 202.36, 202.38, 202.40, 202.42, 202.43, 202.44, 202.47, 202.48, 202.50, 202.51, 202.58, 202.60, 202.62, 202.63, 202.68, 202.70, 202.72, 202.73, 202.74, 202.75, 202.77, 202.78, 202.8, 202.80, 202.81, 202.82, 202.83, 202.84, 202.85, 202.86, 202.87, 202.88, 202.90, 202.91, 202.92, 202.93, 202.94, 202.95, 202.96, 202.97, 202.98, 203, 203.0, 203.00, 203.01, 203.02, 203.10, 203.11, 203.80, 204.00, 204.01, 204.02, 204.1, 204.10, 204.11, 204.12, 204.80, 204.81, 204.90, 204.91, 204.92, 205.00, 205.01, 205.02, 205.1, 205.10, 205.11, 205.12, 205.30, 205.31, 205.80, 205.81, 205.90, 205.91, 206.00, 206.01, 206.02, 206.10, 206.80, 206.81, 206.90, 207.20, 207.21, 207.80, 207.81, 208.0, 208.00, 208.01, 208.02, 208.1, 208.10, 208.11, 208.20, 208.21, 208.80, 208.81, 208.90, 208.91, 208.92, 209.00, 209.01, 209.02, 209.03, 209.10, 209.11, 209.12, 209.13, 209.16, 209.17, 209.20, 209.21, 209.22, 209.23, 209.24, 209.25, 209.26, 209.27, 209.29, 209.30, 209.31, 209.32, 209.33, 209.34, 209.35, 209.36, 209.40, 209.41, 209.43, 209.50, 209.51, 209.52, 209.53, 209.55, 209.56, 209.57, 209.60, 209.61, 209.63, 209.64, 209.65, 209.66, 209.69, 209.70, 209.71, 209.72, 209.73, 209.74, 209.75, 209.79 | C00, C00.0, C00.1, C00.2, C00.3, C00.4, C00.5, C00.6, C00.8, C00.9, C01, C02, C02.0, C02.1, C02.2, C02.3, C02.4, C02.8, C02.9, C03, C03.0, C03.1, C03.9, C04, C04.0, C04.1, C04.8, C04.9, C05, C05.0, C05.1, C05.2, C05.8, C05.9, C06, C06.0, C06.1, C06.2, C06.8, C06.80, C06.89, C06.9, C07, C08, C08.0, C08.1, C08.9, C09, C09.0, C09.1, C09.8, C09.9, C10, C10.0, C10.1, C10.2, C10.3, C10.4, C10.8, C10.9, C11, C11.0, C11.1, C11.2, C11.3, C11.8, C11.9, C12, C13, C13.0, C13.1, C13.2, C13.8, C13.9, C14, C14.0, C14.2, C14.8, C15, C15.3, C15.4, C15.5, C15.8, C15.9, C16, C16.0, C16.1, C16.2, C16.3, C16.4, C16.5, C16.6, C16.8, C16.9, C17, C17.0, C17.1, C17.2, C17.3, C17.8, C17.9, C18, C18.0, C18.1, C18.2, C18.3, C18.4, C18.5, C18.6, C18.7, C18.8, C18.9, C19, C20, C21, C21.0, C21.1, C21.2, C21.8, C22, C22.0, C22.1, C22.2, C22.3, C22.4, C22.7, C22.8, C22.9, C23, C24, C24.0, C24.1, C24.8, C24.9, C25, C25.0, C25.1, C25.2, C25.3, C25.4, C25.7, C25.8, C25.9, C26, C26.0, C26.1, C26.9, C30, C30.0, C30.1, C31, C31.0, C31.1, C31.2, C31.3, C31.8, C31.9, C32, C32.0, C32.1, C32.2, C32.3, C32.8, C32.9, C33, C34, C34.0, C34.00, C34.01, C34.02, C34.1, C34.10, C34.11, C34.12, C34.2, C34.3, C34.30, C34.31, C34.32, C34.8, C34.80, C34.81, C34.82, C34.9, C34.90, C34.91, C34.92, C37, C38, C38.0, C38.1, C38.2, C38.3, C38.4, C38.8, C39, C39.0, C39.9, C40, C40.0, C40.00, C40.01, C40.02, C40.1, C40.10, C40.11, C40.12, C40.2, C40.20, C40.21, C40.22, C40.3, C40.30, C40.31, C40.32, C40.8, C40.80, C40.81, C40.82, C40.9, C40.90, C40.91, C40.92, C41, C41.0, C41.1, C41.2, C41.3, C41.4, C41.9, C43, C43.0, C43.1, C43.10, C43.11, C43.12, C43.2, C43.20, C43.21, C43.22, C43.3, C43.30, C43.31, C43.39, C43.4, C43.5, C43.51, C43.52, C43.59, C43.6, C43.60, C43.61, C43.62, C43.7, C43.70, C43.71, C43.72, C43.8, C43.9, C45, C45.0, C45.1, C45.2, C45.7, C45.9, C46, C46.0, C46.1, C46.2, C46.3, C46.4, C46.5, C46.50, C46.51, C46.52, C46.7, C46.9, C47, C47.0, C47.1, C47.10, C47.11, C47.12, C47.2, C47.20, C47.21, C47.22, C47.3, C47.4, C47.5, C47.6, C47.8, C47.9, C48, C48.0, C48.1, C48.2, C48.8, C49, C49.0, C49.1, C49.10, C49.11, C49.12, C49.2, C49.20, C49.21, C49.22, C49.3, C49.4, C49.5, C49.6, C49.8, C49.9, C49.A, C49.A0, C49.A1, C49.A2, C49.A3, C49.A4, C49.A5, C49.A9, C4A, C4A.0, C4A.1, C4A.10, C4A.11, C4A.12, C4A.2, C4A.20, C4A.21, C4A.22, C4A.3, C4A.30, C4A.31, C4A.39, C4A.4, C4A.5, C4A.51, C4A.52, C4A.59, C4A.6, C4A.60, C4A.61, C4A.62, C4A.7, C4A.70, C4A.71, C4A.72, C4A.8, C4A.9, C50, C50.0, C50.01, C50.011, C50.012, C50.019, C50.02, C50.021, C50.022, C50.029, C50.1, C50.11, C50.111, C50.112, C50.119, C50.12, C50.121, C50.122, C50.129, C50.2, C50.21, C50.211, C50.212, C50.219, C50.22, C50.221, C50.222, C50.229, C50.3, C50.31, C50.311, C50.312, C50.319, C50.32, C50.321, C50.322, C50.329, C50.4, C50.41, C50.411, C50.412, C50.419, C50.42, C50.421, C50.422, C50.429, C50.5, C50.51, C50.511, C50.512, C50.519, C50.52, C50.521, C50.522, C50.529, C50.6, C50.61, C50.611, C50.612, C50.619, C50.62, C50.621, C50.622, C50.629, C50.8, C50.81, C50.811, C50.812, C50.819, C50.82, C50.821, C50.822, C50.829, C50.9, C50.91, C50.911, C50.912, C50.919, C50.92, C50.921, C50.922, C50.929, C51, C51.0, C51.1, C51.2, C51.8, C51.9, C52, C53, C53.0, C53.1, C53.8, C53.9, C54, C54.0, C54.1, C54.2, C54.3, C54.8, C54.9, C55, C56, C56.1, C56.2, C56.9, C57, C57.0, C57.00, C57.01, C57.02, C57.1, C57.10, C57.11, C57.12, C57.2, C57.20, C57.21, C57.22, C57.3, C57.4, C57.7, C57.8, C57.9, C58, C60, C60.0, C60.1, C60.2, C60.8, C60.9, C61, C62, C62.0, C62.00, C62.01, C62.02, C62.1, C62.10, C62.11, C62.12, C62.9, C62.90, C62.91, C62.92, C63, C63.0, C63.00, C63.01, C63.02, C63.1, C63.10, C63.11, C63.12, C63.2, C63.7, C63.8, C63.9, C64, C64.1, C64.2, C64.9, C65, C65.1, C65.2, C65.9, C66, C66.1, C66.2, C66.9, C67, C67.0, C67.1, C67.2, C67.3, C67.4, C67.5, C67.6, C67.7, C67.8, C67.9, C68, C68.0, C68.1, C68.8, C68.9, C69, C69.0, C69.00, C69.01, C69.02, C69.1, C69.10, C69.11, C69.12, C69.2, C69.20, C69.21, C69.22, C69.3, C69.30, C69.31, C69.32, C69.4, C69.40, C69.41, C69.42, C69.5, C69.50, C69.51, C69.52, C69.6, C69.60, C69.61, C69.62, C69.8, C69.80, C69.81, C69.82, C69.9, C69.90, C69.91, C69.92, C70, C70.0, C70.1, C70.9, C71, C71.0, C71.1, C71.2, C71.3, C71.4, C71.5, C71.6, C71.7, C71.8, C71.9, C72, C72.0, C72.1, C72.2, C72.20, C72.21, C72.22, C72.3, C72.30, C72.31, C72.32, C72.4, C72.40, C72.41, C72.42, C72.5, C72.50, C72.59, C72.9, C73, C74, C74.0, C74.00, C74.01, C74.02, C74.1, C74.10, C74.11, C74.12, C74.9, C74.90, C74.91, C74.92, C75, C75.0, C75.1, C75.2, C75.3, C75.4, C75.5, C75.8, C75.9, C76, C76.0, C76.1, C76.2, C76.3, C76.4, C76.40, C76.41, C76.42, C76.5, C76.50, C76.51, C76.52, C76.8, C77, C77.0, C77.1, C77.2, C77.3, C77.4, C77.5, C77.8, C77.9, C78, C78.0, C78.00, C78.01, C78.02, C78.1, C78.2, C78.3, C78.30, C78.39, C78.4, C78.5, C78.6, C78.7, C78.8, C78.80, C78.89, C79, C79.0, C79.00, C79.01, C79.02, C79.1, C79.10, C79.11, C79.19, C79.2, C79.3, C79.31, C79.32, C79.4, C79.40, C79.49, C79.5, C79.51, C79.52, C79.6, C79.60, C79.61, C79.62, C79.7, C79.70, C79.71, C79.72, C79.8, C79.81, C79.82, C79.89, C79.9, C7A, C7A.0, C7A.00, C7A.01, C7A.010, C7A.011, C7A.012, C7A.019, C7A.02, C7A.020, C7A.021, C7A.022, C7A.023, C7A.024, C7A.025, C7A.026, C7A.029, C7A.09, C7A.090, C7A.091, C7A.092, C7A.093, C7A.094, C7A.095, C7A.096, C7A.098, C7A.1, C7A.8, C7B, C7B.0, C7B.00, C7B.01, C7B.02, C7B.03, C7B.04, C7B.09, C7B.1, C7B.8, C80, C80.0, C80.1, C80.2, C81, C81.0, C81.00, C81.01, C81.02, C81.03, C81.04, C81.05, C81.06, C81.07, C81.08, C81.09, C81.1, C81.10, C81.11, C81.12, C81.13, C81.14, C81.15, C81.16, C81.17, C81.18, C81.19, C81.2, C81.20, C81.21, C81.22, C81.23, C81.24, C81.25, C81.26, C81.27, C81.28, C81.29, C81.3, C81.30, C81.31, C81.32, C81.33, C81.34, C81.35, C81.36, C81.37, C81.38, C81.39, C81.4, C81.40, C81.41, C81.42, C81.43, C81.44, C81.45, C81.46, C81.47, C81.48, C81.49, C81.7, C81.70, C81.71, C81.72, C81.73, C81.74, C81.75, C81.76, C81.77, C81.78, C81.79, C81.9, C81.90, C81.91, C81.92, C81.93, C81.94, C81.95, C81.96, C81.97, C81.98, C81.99, C82, C82.0, C82.00, C82.01, C82.02, C82.03, C82.04, C82.05, C82.06, C82.07, C82.08, C82.09, C82.1, C82.10, C82.11, C82.12, C82.13, C82.14, C82.15, C82.16, C82.17, C82.18, C82.19, C82.2, C82.20, C82.21, C82.22, C82.23, C82.24, C82.25, C82.26, C82.27, C82.28, C82.29, C82.3, C82.30, C82.31, C82.32, C82.33, C82.34, C82.35, C82.36, C82.37, C82.38, C82.39, C82.4, C82.40, C82.41, C82.42, C82.43, C82.44, C82.45, C82.46, C82.47, C82.48, C82.49, C82.5, C82.50, C82.51, C82.52, C82.53, C82.54, C82.55, C82.56, C82.57, C82.58, C82.59, C82.6, C82.60, C82.61, C82.62, C82.63, C82.64, C82.65, C82.66, C82.67, C82.68, C82.69, C82.8, C82.80, C82.81, C82.82, C82.83, C82.84, C82.85, C82.86, C82.87, C82.88, C82.89, C82.9, C82.90, C82.91, C82.92, C82.93, C82.94, C82.95, C82.96, C82.97, C82.98, C82.99, C83, C83.0, C83.00, C83.01, C83.02, C83.03, C83.04, C83.05, C83.06, C83.07, C83.08, C83.09, C83.1, C83.10, C83.11, C83.12, C83.13, C83.14, C83.15, C83.16, C83.17, C83.18, C83.19, C83.3, C83.30, C83.31, C83.32, C83.33, C83.34, C83.35, C83.36, C83.37, C83.38, C83.39, C83.5, C83.50, C83.51, C83.52, C83.53, C83.54, C83.55, C83.56, C83.57, C83.58, C83.59, C83.7, C83.70, C83.71, C83.72, C83.73, C83.74, C83.75, C83.76, C83.77, C83.78, C83.79, C83.8, C83.80, C83.81, C83.82, C83.83, C83.84, C83.85, C83.86, C83.87, C83.88, C83.89, C83.9, C83.90, C83.91, C83.92, C83.93, C83.94, C83.95, C83.96, C83.97, C83.98, C83.99, C84, C84.0, C84.00, C84.01, C84.02, C84.03, C84.04, C84.05, C84.06, C84.07, C84.08, C84.09, C84.1, C84.10, C84.11, C84.12, C84.13, C84.14, C84.15, C84.16, C84.17, C84.18, C84.19, C84.4, C84.40, C84.41, C84.42, C84.43, C84.44, C84.45, C84.46, C84.47, C84.48, C84.49, C84.6, C84.60, C84.61, C84.62, C84.63, C84.64, C84.65, C84.66, C84.67, C84.68, C84.69, C84.7, C84.70, C84.71, C84.72, C84.73, C84.74, C84.75, C84.76, C84.77, C84.78, C84.79, C84.9, C84.90, C84.91, C84.92, C84.93, C84.94, C84.95, C84.96, C84.97, C84.98, C84.99, C84.A, C84.A0, C84.A1, C84.A2, C84.A3, C84.A4, C84.A5, C84.A6, C84.A7, C84.A8, C84.A9, C84.Z, C84.Z0, C84.Z1, C84.Z2, C84.Z3, C84.Z4, C84.Z5, C84.Z6, C84.Z7, C84.Z8, C84.Z9, C85, C85.1, C85.10, C85.11, C85.12, C85.13, C85.14, C85.15, C85.16, C85.17, C85.18, C85.19, C85.2, C85.20, C85.21, C85.22, C85.23, C85.24, C85.25, C85.26, C85.27, C85.28, C85.29, C85.8, C85.80, C85.81, C85.82, C85.83, C85.84, C85.85, C85.86, C85.87, C85.88, C85.89, C85.9, C85.90, C85.91, C85.92, C85.93, C85.94, C85.95, C85.96, C85.97, C85.98, C85.99, C88, C88.0, C88.2, C88.3, C88.4, C88.8, C88.9, C90, C90.0, C90.00, C90.01, C90.02, C90.1, C90.10, C90.11, C90.12, C90.2, C90.20, C90.21, C90.22, C90.3, C90.30, C90.31, C90.32, C91, C91.0, C91.00, C91.01, C91.02, C91.1, C91.10, C91.11, C91.12, C91.3, C91.30, C91.31, C91.32, C91.4, C91.40, C91.41, C91.42, C91.5, C91.50, C91.51, C91.52, C91.6, C91.60, C91.61, C91.62, C91.9, C91.90, C91.91, C91.92, C91.A, C91.A0, C91.A1, C91.A2, C91.Z, C91.Z0, C91.Z1, C91.Z2, C92, C92.0, C92.00, C92.01, C92.02, C92.1, C92.10, C92.11, C92.12, C92.2, C92.20, C92.21, C92.22, C92.3, C92.30, C92.31, C92.32, C92.4, C92.40, C92.41, C92.42, C92.5, C92.50, C92.51, C92.52, C92.6, C92.60, C92.61, C92.62, C92.9, C92.90, C92.91, C92.92, C92.A, C92.A0, C92.A1, C92.A2, C92.Z, C92.Z0, C92.Z1, C92.Z2, C93, C93.0, C93.00, C93.01, C93.02, C93.1, C93.10, C93.11, C93.12, C93.3, C93.30, C93.31, C93.32, C93.9, C93.90, C93.91, C93.92, C93.Z, C93.Z0, C93.Z1, C93.Z2, C94, C94.0, C94.00, C94.01, C94.02, C94.2, C94.20, C94.21, C94.22, C94.3, C94.30, C94.31, C94.32, C94.4, C94.40, C94.41, C94.42, C94.6, C94.8, C94.80, C94.81, C94.82, C95, C95.0, C95.00, C95.01, C95.02, C95.1, C95.10, C95.11, C95.12, C95.9, C95.90, C95.91, C95.92, C96, C96.0, C96.2, C96.4, C96.5, C96.6, C96.9, C96.A, C96.Z |

# Table B. CPT codes used for MRI Brain

| **Code** | **Description** | **Type** |
| --- | --- | --- |
| 70551 | MR BRAIN | CPT4 |
| 70551 | MRI (EG, PROTON), BRAIN (INCLUDING BRAIN STEM) | CPT4 |
| 70553 | MRI (EG, PROTON), WITHOUT CONT | CPT4 |
| 70552 | MR BRAIN W/CONT | CPT4 |
| 70553 | MAGNETIC IMAGE, BRAIN (MRI) | CPT4 |
| 70552 | MRI (EG, PROTON), BRAIN (INCLUDING BRAIN STEM) | CPT4 |

# Table C. Data elements from GNSIS database included in the study

| Variable Name | Variable Type | Unit/Format/Levels | Variable Description |
| --- | --- | --- | --- |
| Patient ID | String | Format: PT##### | Unique ID assigned to de-identified patients |
| Patient Sex/Gender | Categorical | Levels: Female Male | Patient's Sex/Gender as recorded in patient's Electronic Health Records (EHR). |
| Patient's Birth Date | Date | Format: YYYY-MM-DD | Patient's date of birth as recorded in the patient's EHR. |
| Patient's Death Date | Date | YYYY-MM-DD   (NA if the patient is alive or date of death has been removed or not recorded in EHR) | Patient's date of death as recorded in the patient's EHR.  Comments: During data-pull and de-identification, the Date of Death was masked (changed to NA) if the patient was older than 89 years old. |
| Last Active Date | Date | YYYY-MM-DD | Date of last active date of the patient in the Geisinger System; includes any kind of encounter as recorded in the EHR (as of 2019-05-24). Compiled from the patient's last encounter and last lab dates. |
| Family History of Heart Disorder | Categorical | Levels: 0 = Absent 1 = Present | Family history of the patient for a Heart disorder as recorded in the EHR; taken as absent if not mentioned in EHR. |
| Family History of Stroke | Categorical | Levels: 0 = Absent 1 = Present | Family history of the patient for Stroke as recorded in the EHR; taken as absent if not mentioned in EHR. |
| Atrial Fibrillation or Flutter (at index) | Categorical | Levels: 0 = Absent 1 = Present | Past medical history of Atrial Fibrillation or Flutter till the Index Stroke Date (including Index Stroke Date). |
| Hypertension (at index) | Categorical | Levels: 0 = Absent 1 = Present | Past medical history of Hypertension till the Index Stroke Date (including Index Stroke Date); as recorded in the EHR; taken as absent if not mentioned in EHR. |
| Myocardial Infarction (at index) | Categorical | Levels: 0 = Absent 1 = Present | Past medical history of Myocardial Infarction till the Index Stroke Date (including Index Stroke Date); as recorded in the EHR; taken as absent if not mentioned in EHR. |
| Diabetes (at index) | Categorical | Levels: 0 = Absent 1 = Present | Past medical history of Diabetes till the Index Stroke Date (including Index Stroke Date); as recorded in the EHR; taken as absent if not mentioned in EHR. |
| Dyslipidemia (at index) | Categorical | Levels: 0 = Absent 1 = Present | Past medical history of Dyslipidemia till the Index Stroke Date (including Index Stroke Date); as recorded in the EHR; taken as absent if not mentioned in EHR. |
| Heart Failure (at index) | Categorical | Levels: 0 = Absent 1 = Present | Past medical history of Heart failure till the Index Stroke Date (including Index Stroke Date); as recorded in the EHR; taken as absent if not mentioned in EHR. |
| Hypercoagulable States (at index) | Categorical | Levels: 0 = Absent 1 = Present | Past medical history of Hypercoagulable States till the Index Stroke Date (including Index Stroke Date); as recorded in the EHR; taken as absent if not mentioned in EHR. |
| Chronic Liver Disease (at index) | Categorical | Levels: 0 = Absent 1 = Present | Past medical history of Chronic Liver Disease till the Index Stroke Date (including Index Stroke Date) |
| Chronic Lung Diseases (at index) | Categorical | Levels: 0 = Absent 1 = Present | Past medical history of Chronic Lung Diseases till the Index Stroke Date (including Index Stroke Date); as recorded in the EHR; taken as absent if not mentioned in EHR. |
| Rheumatic Diseases (at index) | Categorical | Levels: 0 = Absent 1 = Present | Past medical history of Rheumatic Diseases till the Index Stroke Date (including Index Stroke Date); as recorded in the EHR; taken as absent if not mentioned in EHR. |
| Chronic Kidney Disease (at index) | Categorical | Levels: 0 = Absent 1 = Present | Past medical history of Chronic Kidney Disease till the Index Stroke Date (including Index Stroke Date); as recorded in the EHR; taken as absent if not mentioned in EHR. |
| Neoplasm (at index) | Categorical | Levels: 0 = Absent 1 = Present | Past medical history of Neoplasm till the Index Stroke Date (including Index Stroke Date); as recorded in the EHR; taken as absent if not mentioned in EHR. |
| Peripheral Vascular Disease (at index) | Categorical | Levels: 0 = Absent 1 = Present | Past medical history of Peripheral Vascular Disease till the Index Stroke Date (including Index Stroke Date); as recorded in the EHR; taken as absent if not mentioned in EHR. |
| Past Ischemic Stroke (at index) | Categorical | Levels: 0 = Absent 1 = Present | Past medical history of Ischemic Stroke till the Index Stroke Date (including Index Stroke Date); as recorded in the EHR; taken as absent if not mentioned in EHR.  Comment: On the index stroke date, only non-primary diagnoses were considered. |
| Past Hemorrhagic Stroke (at index) | Categorical | Levels: 0 = Absent 1 = Present | Past medical history of Hemorrhagic Stroke till the Index Stroke Date (including Index Stroke Date); as recorded in the EHR; taken as absent if not mentioned in EHR. |
| Index Stroke Date | Date | YYYY-MM-DD | Date of the first encounter at Geisinger in which:  a) primary diagnosis was an ischemic stroke, and b) MRI was available in the encounter, and c) length of stay was at least 24 hours |
| Encounter Type | Categorical | Levels: ED ONLY: Admission to the Emergency department only, ED TO IP: Admission to the Emergency Department and then to Inpatient department, IP ONLY: Admission to Inpatient department | The department in which the patient was admitted during the Index Stroke Date |
| ICD Code at Index Stroke Date | String |  | ICD-9-CM or ICD-10-CM codes used for the primary diagnosis |
| Name of the ICD Code at Index Stroke Date | String |  | Corresponding primary diagnosis based on the ICD code used |
| Patient's Age at Index Stroke Date | Numerical | Unit: Years | Age of the patient at the Index Stroke Date; determined from Patient's Birthdate and Index Stroke Date  Comments: Changed to 89 if value greater than 89 as part of de-identification |
| NIHSS Score at the Index Stroke Event | Integer | Unit: NA | NIH Stroke Scale (NIHSS) score of the patient at the Index Stroke Event |
| Recurrence of Stroke | Categorical | Levels: 0 = Absent 1 = Present | Whether the patient had a stroke recurrence or not.  Based on whether the stroke ICD codes were present in primary diagnosis in the subsequent encounters to Geisinger, MRI, and duration of admission criteria. |
| Date of Recurrence of Stroke | Date | YYYY-MM-DD  NA (for non-recurrent stroke patients) | The date on which recurrence of stroke occurred as recorded in EHR |
| ICD Code for Recurrence of Stroke | String |  | ICD-9 and ICD-10 codes used for the primary diagnosis |
| Name of the ICD Code for Recurrence of Stroke | String |  | Corresponding primary diagnosis based on the ICD code used |
| Median BMI of the patient before Index Stroke Date | Numeric | Unit: Kg/m^2 | Median BMI of the patient in the three years leading to the index stroke event |

# Table D. Comparison of Age, Sex and NIHSS between excluded patients with missing BMI and patients included in the analysis

|  | **BMI Missing** | **BMI present** | **p** |
| --- | --- | --- | --- |
| n | 852 | 6703 |  |
| Age at index stroke (mean (SD)) | 69.76 (13.84) | 70.23 (13.47) | 0.344 |
| Age at index stroke (median [IQR]) | 71.65 [59.60, 81.00] | 71.50 [61.05, 81.20] | 0.436 |
| Male, n (%) | 416 (48.8) | 3476 (51.9) | 0.103 |
| NIHSS (median [IQR]) | 3.50 [2.00, 7.75] | 3.00 [2.00, 6.00] | 0.535 |

# Table E. p-values for pairwise Log-rank test for survival at one year for four BMI categories

|  | Overall | Women | Men |
| --- | --- | --- | --- |
| Normal and Underweight | 0.9892 | 0.32 | 0.09711 |
| Normal and Overweight | <0.0001 | <0.0001 | <0.0001 |
| Normal and Obese | <0.0001 | <0.0001 | <0.0001 |
| Underweight and Overweight | 0.0071 | 0.31 | 0.0008 |
| Underweight and Obese | 0.0002 | 0.11 | <0.0001 |
| Overweight and Obese | 0.0087 | 0.13 | 0.02792 |

# Table F. p-values for pairwise log-rank test for survival at one year for three BMI categories

|  | Overall | Women | Men |
| --- | --- | --- | --- |
| Non-overweight and Overweight | <0.0001 | <0.0001 | <0.0001 |
| Non-overweight and Obese | <0.0001 | <0.0001 | <0.0001 |
| Overweight and Obese | 0.0072 | 0.086 | 0.023 |

# Table G. Multivariate stratified Cox proportional hazards model on 1-year mortality for patients with NIHSS data (N=1,782)

| **Variable** | **HR** | **95% Confidence Interval** | **p-value** |
| --- | --- | --- | --- |
| **BMI Categories:** |  |  |  |
| Non-overweight | Reference |  |  |
| Overweight | 0.58 | 0.41 - 0.81 | 0.001 |
| Obese | 0.51 | 0.35 - 0.73 | 0.000 |
| **Age at ischemic stroke** | 1.04 | 1.03 - 1.06 | 0.000 |
| **Heart failure** | 2.32 | 1.68 - 3.21 | 0.000 |
| **Neoplasm** | 1.61 | 1.16 - 2.22 | 0.004 |
| **Myocardial Infarction** | 1.56 | 1.08 - 2.26 | 0.018 |
| **Diabetes** | 1.29 | 0.95 - 1.76 | 0.100 |
| **Dyslipidemia** | 0.80 | 0.59 - 1.08 | 0.144 |
| **Rheumatic diseases** | 1.46 | 0.87 - 2.44 | 0.150 |
| **Atrial fibrillation/flutter** | 1.18 | 0.87 - 1.61 | 0.277 |
| **Chronic lung diseases** | 0.94 | 0.68 - 1.29 | 0.687 |
| **Chronic kidney disease** | 1.04 | 0.76 - 1.42 | 0.822 |
| **Gender:** |  |  |  |
| Female | Reference |  |  |
| Male | 0.98 | 0.74 - 1.31 | 0.904 |

***NIHSS and peripheral vascular disease were used as strata terms in the model**

# Table H. Assessment of Schoenfeld residuals for the stratified Cox model for all patients in the study

|  | chisq | df | p |
| --- | --- | --- | --- |
| BMI Category | 1.2766406 | 2 | 0.5281789 |
| Age at index stroke | 0.4020228 | 1 | 0.5260464 |
| Myocardial infarction | 1.4619119 | 1 | 0.2266262 |
| Atrial fibrillation/flutter | 0.9528393 | 1 | 0.3289976 |
| Heart Failure | 0.1183393 | 1 | 0.7308426 |
| Neoplasm | 2.3854724 | 1 | 0.1224679 |
| Chronic lung diseases | 2.6689052 | 1 | 0.1023264 |
| Gender | 0.4868196 | 1 | 0.4853494 |
| Diabetes | 2.9624502 | 1 | 0.0852187 |
| GLOBAL | 13.0332709 | 10 | 0.2218175 |

# Table I. Assessment of Schoenfeld residuals for stratified Cox model patients with NIHSS data

|  | chisq | df | p |
| --- | --- | --- | --- |
| BMI Categories | 0.1637364 | 2 | 0.9213934 |
| Age at index stroke | 2.7487580 | 1 | 0.0973300 |
| Myocardial Infarction | 3.0963930 | 1 | 0.0784660 |
| Atrial fibrillation/flutter | 0.6391498 | 1 | 0.4240188 |
| Heart failure | 0.0762327 | 1 | 0.7824693 |
| Chronic kidney disease | 0.7060664 | 1 | 0.4007528 |
| Neoplasm | 0.3278887 | 1 | 0.5669050 |
| Dyslipidemia | 0.0000483 | 1 | 0.9944528 |
| Rheumatic diseases | 2.1453636 | 1 | 0.1430012 |
| Chronic lung diseases | 0.2887222 | 1 | 0.5910406 |
| Gender | 0.0026276 | 1 | 0.9591180 |
| Diabetes | 1.5206980 | 1 | 0.2175139 |
| GLOBAL | 12.8737662 | 13 | 0.4576090 |

# Table J. Multivariate logistic regression on one-year mortality for patients with at least one-year follow-up data

| Variable | OR | 95% Confidence Interval | p-value |
| --- | --- | --- | --- |
| Age at ischemic stroke | 1.04 | 1.03 - 1.05 | < 0.001 |
| BMI Categories: |  |  |  |
| Non-overweight | Reference |  |  |
| Overweight | 0.54 | 0.45 - 0.65 | < 0.001 |
| Obese | 0.50 | 0.41 - 0.60 | < 0.001 |
| Neoplasm | 1.76 | 1.47 - 2.09 | < 0.001 |
| Heart failure | 1.86 | 1.52 - 2.26 | < 0.001 |
| Dyslipidemia | 0.61 | 0.52 - 0.71 | < 0.001 |
| Chronic kidney disease | 1.61 | 1.34 - 1.93 | < 0.001 |
| Diabetes | 1.30 | 1.10 - 1.53 | 0.002 |
| Atrial fibrillation/flutter | 1.23 | 1.04 - 1.47 | 0.017 |
| Myocardial infarction | 1.30 | 1.04 - 1.62 | 0.018 |
| Rheumatic diseases | 1.42 | 1.02 - 1.95 | 0.033 |
| Peripheral vascular disease | 1.11 | 0.91 - 1.34 | 0.289 |
| Chronic lung diseases | 1.06 | 0.89 - 1.27 | 0.493 |
| Gender: |  |  |  |
| Female | Reference |  |  |
| Male | 1.02 | 0.88 - 1.20 | 0.771 |

# Table K. Summary of different studies regarding Obesity and Stroke Mortality

| Study | Year | Sample Size | Results/Conclusion |
| --- | --- | --- | --- |
| Olsen *et al*.^1^ | 2008 | 21,884 | Hazard ratio (HR) in overweight and obese categories significantly decreased but no significant difference in severely obese patients. |
| Towfighi *et al*.^2^ | 2009 | 644 | Higher BMI has a protective effect on older patients, but higher BMI is associated with increased risk of death in younger individuals. |
| Kim *et al.*^3^ | 2011 | 1,356 | No significant difference in 30-day mortality; Significant decrease in HR in overweight and obese patients (in hemorrhagic stroke patients) in the long term. |
| Ryu *et al.*^4^ | 2011 | 1,592 | HR in underweight patients significantly higher; HR in overweight and obese patients not significantly decreased. |
| Vemmos *et al.*^5^ | 2011 | 2,785 | Obese and overweight patients have significantly better long-term survival compared to normal BMI. |
| Kim *et al.*^6^ | 2012 | 34,132 | Inverse relationship between obesity and mortality became evident at 90 days and significant at 1 year from stroke. |
| Andersen *et al.*^7^ | 2013 | 29,326 | Obesity was associated with reduced mortality compared to normal weight patients. |
| Doehner *et al*.^8^ | 2013 | 1,521 | Obese and overweight patients with stroke or TIA had better survival compared to BMI <25. |
| Bell *et al*.^9^ | 2013 | 3,173 | Significant HR decrease in overweight and obese older women with stroke; HR in underweight group non-significant. |
| Dehlendorff *et al.*^10^ | 2014 | 53,812 | No evidence of the obesity paradox. Stroke occurred at a significantly younger age for patients with higher BMI. |
| Barba *et al.*^11^ | 2015 | 201,272 | Obesity in patients hospitalized with stroke was associated with lower in-hospital mortality. |
| Aparicio *et al.*^13^ | 2017 | 782 | Overweight and mildly obese stroke patients have better 10-year survival compared to normal-weight patients. |
| Choi *et al.*^14^ | 2019 | 3,599 | Obesity is associated with lower mortality risk specially in elderly patients. |

# Table L. New diagnoses of comorbidities after index stroke event for the overall study population

|  | **Past History**  **At Index Date** | **New Diagnosis**  **Between Index Date and Last Active Date (or 365 days whichever is smaller)** |
| --- | --- | --- |
| N | 6703 | 6703 |
| Atrial fibrillation/flutter | 1440 (21.5) | 276 (4.1) |
| Hypertension | 5260 (78.5) | 303 (4.5) |
| Myocardial infarction | 763 (11.4) | 180 (2.7) |
| Diabetes | 2236 (33.4) | 114 (1.7) |
| Dyslipidemia | 4380 (65.3) | 596 (8.9) |
| Heart Failure | 900 (13.4) | 228 (3.4) |
| Hypercoagulable states | 88 (1.3) | 58 (0.9) |
| Chronic Lung diseases | 1538 (22.9) | 171 (2.6) |
| Rheumatic diseases | 287 (4.3) | 21 (0.3) |
| Chronic Kidney disease | 1293 (19.3) | 262 (3.9) |
| Neoplasm | 1119 (16.7) | 133 (2.0) |
| Peripheral vascular disease | 1094 (16.3) | 334 (5.0) |
| Hemorrhagic stroke | 0 (0.0) | 112 (1.7) |

# Table M. New diagnoses of comorbidities after index stroke event for the three BMI groups

|  | **Past History**  **At Index Date** | | | | **New Diagnosis**  **Between Index Date and Last Active Date (or 365 days whichever is smaller)** | | | |
| --- | --- | --- | --- | --- | --- | --- | --- | --- |
|  | Non-overweight | Overweight | Obese | p | Non-overweight | Overweight | Obese | p |
| n | 1638 | 2298 | 2767 |  | 1638 | 2298 | 2767 |  |
| Atrial fibrillation/flutter | 375 (22.9) | 494 (21.5) | 571 (20.6) | 0.211 | 64 (3.9) | 97 (4.2) | 115 (4.2) | 0.880 |
| Hypertension | 1185 (72.3) | 1772 (77.1) | 2303 (83.2) | <0.001 | 74 (4.5) | 104 (4.5) | 125 (4.5) | 1.000 |
| Myocardial infarction | 175 (10.7) | 262 (11.4) | 326 (11.8) | 0.540 | 53 (3.2) | 57 (2.5) | 70 (2.5) | 0.283 |
| Diabetes | 309 (18.9) | 687 (29.9) | 1240 (44.8) | <0.001 | 19 (1.2) | 38 (1.7) | 57 (2.1) | 0.081 |
| Dyslipidemia | 921 (56.2) | 1529 (66.5) | 1930 (69.8) | <0.001 | 142 (8.7) | 207 (9.0) | 247 (8.9) | 0.931 |
| Heart Failure | 220 (13.4) | 277 (12.1) | 403 (14.6) | 0.033 | 58 (3.5) | 73 (3.2) | 97 (3.5) | 0.763 |
| Hypercoagulable states | 26 (1.6) | 24 (1.0) | 38 (1.4) | 0.315 | 10 (0.6) | 20 (0.9) | 28 (1.0) | 0.380 |
| Chronic Lung diseases | 427 (26.1) | 474 (20.6) | 637 (23.0) | <0.001 | 55 (3.4) | 45 (2.0) | 71 (2.6) | 0.023 |
| Rheumatic diseases | 88 (5.4) | 90 (3.9) | 109 (3.9) | 0.043 | 7 (0.4) | 7 (0.3) | 7 (0.3) | 0.603 |
| Chronic Kidney disease | 288 (17.6) | 417 (18.1) | 588 (21.3) | 0.003 | 53 (3.2) | 85 (3.7) | 124 (4.5) | 0.097 |
| Neoplasm | 306 (18.7) | 433 (18.8) | 380 (13.7) | <0.001 | 40 (2.4) | 50 (2.2) | 43 (1.6) | 0.089 |
| Peripheral vascular disease | 291 (17.8) | 414 (18.0) | 389 (14.1) | <0.001 | 81 (4.9) | 109 (4.7) | 144 (5.2) | 0.752 |
| Hemorrhagic stroke | 0 (0.0) | 0 (0.0) | 0 (0.0) | NaN | 34 (2.1) | 36 (1.6) | 42 (1.5) | 0.336 |

# Figure A. Flow-chart of inclusion-exclusion of subjects in the study


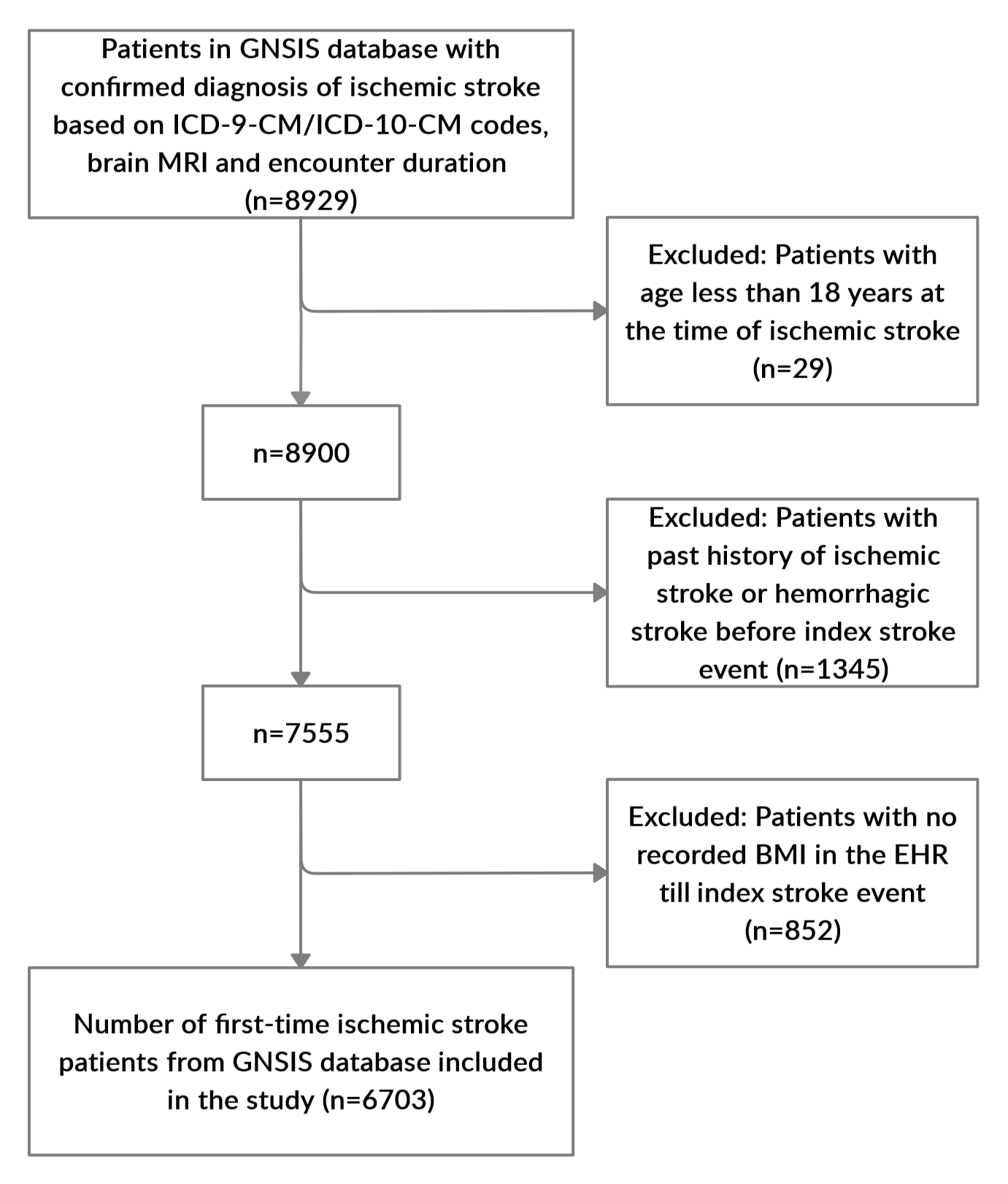


# Figure B. Margin plots of missing BMI with respect to Age, Sex and NIHSS


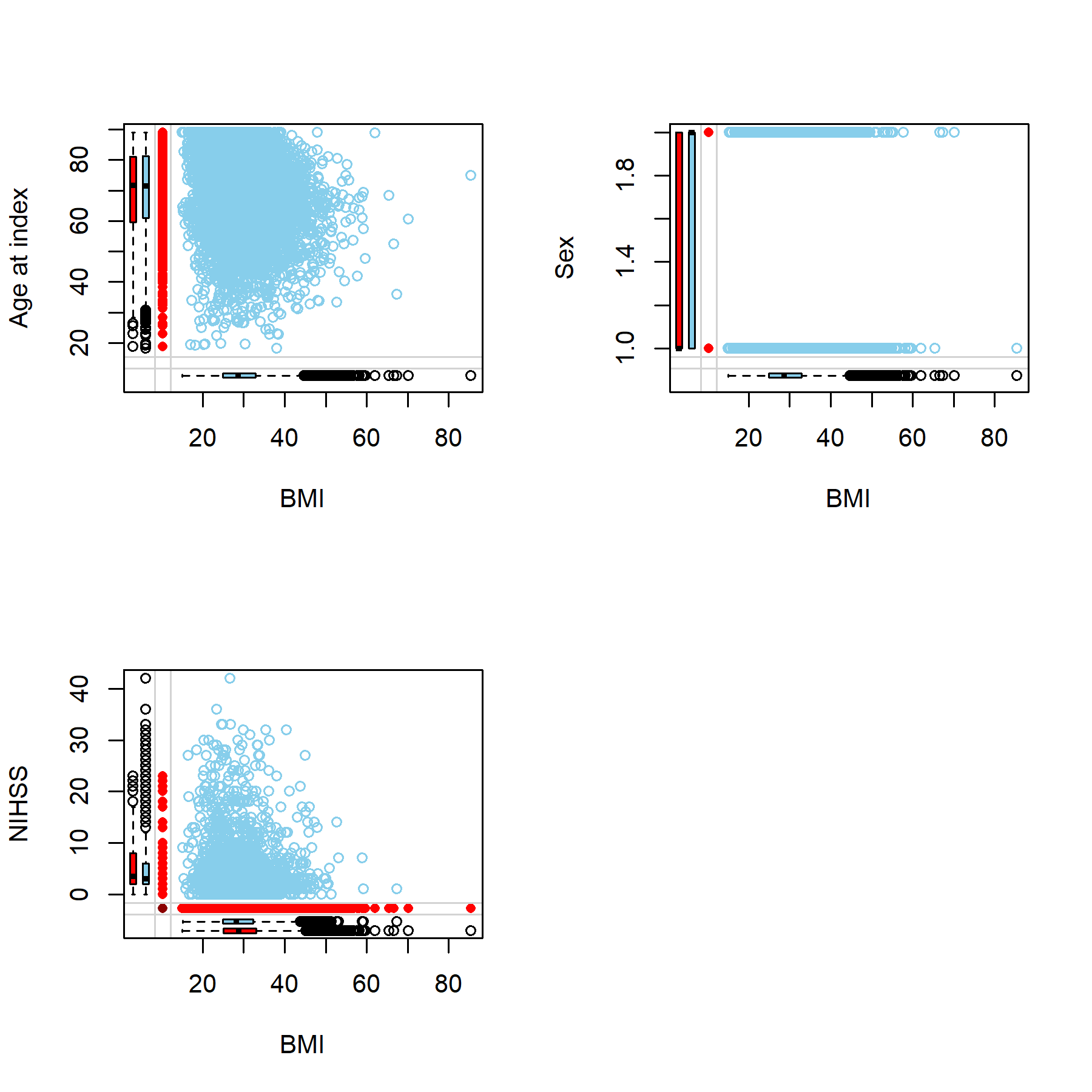


References:

1. Olsen TS, Dehlendorff C, Petersen HG, Andersen KK. Body mass index and poststroke mortality. *Neuroepidemiology*. 2008;30(2):93-100. doi:10.1159/000118945

2. Towfighi A, Ovbiagele B. The impact of body mass index on mortality after stroke. *Stroke*. 2009;40(8):2704-2708. doi:10.1161/STROKEAHA.109.550228

3. Kim BJ, Lee SH, Ryu WS, Kim CK, Lee J, Yoon BW. Paradoxical longevity in obese patients with intracerebral hemorrhage. *Neurology*. 2011;76(6):567-573. doi:10.1212/WNL.0b013e31820b7667

4. Ryu WS, Lee SH, Kim CK, Kim BJ, Yoon BW. Body mass index, initial neurological severity and long-term mortality in ischemic stroke. *Cerebrovasc Dis*. 2011;32(2):170-176. doi:10.1159/000328250

5. Vemmos K, Ntaios G, Spengos K, et al. Association between obesity and mortality after acute first-ever stroke: The obesity-stroke paradox. *Stroke*. 2011;42(1):30-36. doi:10.1161/STROKEAHA.110.593434

6. Kim BJ, Lee SH, Jung KH, Yu KH, Lee BC, Roh JK. Dynamics of obesity paradox after stroke, related to time from onset, age, and causes of death. *Neurology*. 2012;79(9):856-863. doi:10.1212/WNL.0b013e318266fad1

7. Andersen KK, Olsen TS. The obesity paradox in stroke: Lower mortality and lower risk of readmission for recurrent stroke in obese stroke patients. *Int J Stroke*. 2015;10(1):99-104. doi:10.1111/ijs.12016

8. Doehner W, Schenkel J, Anker SD, Springer J, Audebert H. Overweight and obesity are associated with improved survival, functional outcome, and stroke recurrence after acute stroke or transient ischaemic attack: Observations from the tempis trial. *Eur Heart J*. 2013;34(4):268-277. doi:10.1093/eurheartj/ehs340

9. Bell CL, Lacroix A, Masaki K, et al. Prestroke factors associated with poststroke mortality and recovery in older women in the women’s health initiative. *J Am Geriatr Soc*. 2013;61(8):1324-1330. doi:10.1111/jgs.12361

10. Dehlendorff C, Andersen KK, Olsen TS. Body mass index and death by stroke no obesity paradox. *JAMA Neurol*. 2014;71(8):978-984. doi:10.1001/jamaneurol.2014.1017

11. Barba R, Marco J, Ruiz J, et al. The obesity paradox in stroke: Impact on mortality and short-term readmission. *J Stroke Cerebrovasc Dis*. 2015;24(4):766-770. doi:10.1016/j.jstrokecerebrovasdis.2014.11.002

12. Wohlfahrt P, Lopez-Jimenez F, Krajcoviechova A, et al. The obesity paradox and survivors of ischemic stroke. *J Stroke Cerebrovasc Dis*. 2015;24(6):1443-1450. doi:10.1016/j.jstrokecerebrovasdis.2015.03.008

13. Aparicio HJ, Himali JJ, Beiser AS, et al. Overweight, Obesity, and Survival After Stroke in the Framingham Heart Study. *J Am Heart Assoc*. 2017;6(6). doi:10.1161/JAHA.116.004721

14. Choi H, Nam HS, Han E. Body mass index and clinical outcomes in patients after ischaemic stroke in South Korea: A retrospective cohort study. *BMJ Open*. 2019;9(8):e028880. doi:10.1136/bmjopen-2018-028880
