# Supplementary material for: Obesity and mortality after the first ischemic stroke: Is obesity paradox real?
Source: PLoS One. 2021 Feb 10;16(2):e0246877. doi: 10.1371/journal.pone.0246877 (PMC7875337; doi:10.1371/journal.pone.0246877)
Supplement: Attachment — Submitted filename: Response to Reviewers.docx [file pone.0246877.s002.docx]

January 15, 2021

Aristeidis H. Katsanos, MD, PhD

Academic Editor

PLOS ONE

Dear Dr. Katsanos,

We would like to thank you for inviting us to submit a revised draft of our manuscript titled “Obesity and mortality after the first ischemic stroke: Is obesity paradox real?” to PLOS ONE.

We would also like to thank you and the reviewers for your time and effort to provide us with your valuable feedback on this paper. We have made changes to the manuscript to reflect the suggestions that you have provided. We hope our edits and responses below are able to address your concerns.

**Journal Requirements:**

*Response: The manuscript has been edited to meet PLOS ONE’s style requirements. A revised cover letter has been submitted to address data availability, funding statement and competing interests statement. ORCID iD is available for the corresponding author. Captions for Supporting Information files have been added at the end of the manuscript.*

**Reviewer 1 comments:**

This is a retrospective, cohort study of consecutive first-ever ischemic stroke patients with the aim to investigate the association between baseline BMI and all-cause mortality after stroke. This is a well-designed study, based on high quality data derived from GNSIS database, assessed with robust statistical analyses, producing well-presented results supporting the obesity paradox which has been questioned in previous studies. What is more, the authors have already adequately discussed all potential limitations.

For further discussion and due to the existence of data of such a high quality, it would be of interest if an association could be presented between the obesity and mortality among specific ischemic stroke subtypes.

***Response: Thank you for the comment. Specific ischemic stroke subtypes based on TOAST criteria are not part of the structured data of GNSIS database at the present. An association between obesity and mortality among different stroke subtypes is certainly interesting and we will strive to see if it can be done in the future.***

**Reviewer 2 comments:**

The study by Chaudhary et al. provides evidence in support of the “obesity paradox” in ischemic stroke patients. In this retrospective cohort of 6,703 first-time ischemic stroke patients, overweight and obese patients were found to have statistically decreased hazard ratio for one-year mortality in comparison to non-overweight patients. The manuscript is very well written, the methods and statistical analyses are adequately presented. The authors could consider few suggestions that might further improve the results/discussion.

1. Are underlying stroke etiologies captured in the GNSIS database? Considering the noted differences in cardiovascular risk factors in obese/overweight vs. non-overweight patients (as shown in Table 1), it would be interesting to assess whether small vessel disease or other underlying stroke etiologies might differ between obese/overweight vs. non-overweight patients (i.e., with subsequent implications in the functional recovery after stroke).

***Response: We appreciate the comment. Stroke etiology and subclassification based on TOAST criteria is not part of the GNSIS database at the moment. Differences in stroke etiology between obese/overweight vs. non-overweight patients is very interesting and may be pursued in the future if possible***

2. Could the authors discuss on whether ischemic/hypoxic preconditioning might be associated with improved functional outcome in obese/overweight patients? Can the rates of concomitant Obesity hypoventilation syndrome (OHS) be retrieved for included patients?

***Response: Thank you for much for bringing up “ischemic/hypoxic preconditioning” as a possible explanation for improved outcome in obese/overweight patients. A section on this phenomenon has been added to the discussion [Manuscript Line 266-274].***

***The investigators explored Obesity Hypoventilation Syndrome (OHS) in the GNSIS database. Only 15 obese patients out of 6,703 patients included in this study had this particular diagnosis. Of these 15 patients, 5 died within 1 year, 7 were alive at 1 year and 3 were censored before 1 year. Due to the small number of patients with this diagnosis and all of them being obese, further analysis could not be done. This is partly because diagnosis of OHS requires BMI of more than 30 kg/m2 and alveolar hypoventilation which cannot be attributed to other conditions and thus this diagnosis seems to be uncommon.***

***We also explored obstructive sleep apnea (OSA) rates in our study participants. OSA rate in non-overweight, overweight and obese patients was 1.4%, 3.1% and 11.5% respectively. OSA was not associated with one-year all-cause mortality and its inclusion in the stratified cox model did not change our results.***

3. Minor – Line 256, p 12: “has” should be corrected to “have”.

***Response: Thank you for the comment. It has been corrected in the manuscript.***

We would like to thank you once again for the opportunity to strengthen our manuscript. We hope these revisions are adequate according to your requirements.

Sincerely,

**Ramin Zand, MD, MPH**

Associate Professor of Neurology and Medicine

Director, Stroke Program, Geisinger Northeast Region

Chairman, GRA Stroke Task Force, American Heart Association

Ph: 570-808-7330 | Fax: 570-808-3209 | ramin.zand@gmail.com | rzand@geisinger.edu

Geisinger Neuroscience Institute

Geisinger Health System

100 N Academy Ave

Danville, PA 17822

**On behalf of co-authors**
